# Long-lasting insecticide-treated bed net ownership and use among children under five years of age following a targeted distribution in central Uganda

**DOI:** 10.1186/1475-2875-13-185

**Published:** 2014-05-19

**Authors:** Humphrey Wanzira, Adoke Yeka, Ruth Kigozi, Denis Rubahika, Sussann Nasr, Asadu Sserwanga, Moses Kamya, Scott Filler, Grant Dorsey, Laura Steinhardt

**Affiliations:** 1 Infectious Diseases Research Collaboration, Kampala, Uganda; 2 President’s Malaria Initiative, Malaria Branch, Centers for Disease Control and Prevention, Luanda, Angola; 3 Uganda National Malaria Control Programme, Ministry of Health, Kampala, Uganda; 4 Makerere University, Kampala, Uganda; 5 The Global Fund to Fight AIDS, Tuberculosis, and Malaria, Geneva, Switzerland; 6 University of California, San Francisco, CA, USA; 7 Malaria Branch, Division of Parasitic Diseases and Malaria, Center for Global Health, Centers for Disease Control and Prevention, Atlanta, GA, USA

**Keywords:** Malaria, Long-lasting insecticide-treated bed nets, Intervention coverage

## Abstract

**Background:**

Universal coverage of long-lasting insecticide-treated bed nets (LLINs) for prevention of malaria was adopted by the Uganda National Malaria Control Programme in 2007. The first mass distribution of LLINs was implemented in 2010. Initially, a campaign targeted to households with pregnant women and children aged <five years was carried out, prior to a planned fill-in campaign to achieve universal LLIN coverage. This survey was conducted after the targeted distribution in central Uganda to assess progress in LLIN ownership and usage among children <five years.

**Methods:**

A two-stage, cluster-sample, cross-sectional household survey was carried out in early 2011 in Central region districts surveyed during the 2009 Malaria Indicator Survey (MIS). In the first sampling stage, 30 enumeration areas (EAs) were selected and all households were enumerated. Within each sampled EA, 20 households were randomly selected for interview using two questionnaires: a household questionnaire and a woman’s questionnaire for all women aged 15-49 years, both modified from the MIS.

**Results:**

When compared to 2009 MIS results, household ownership of at least one LLIN increased by 47%, from 22 to 69% after the targeted campaign. LLIN use among children <five years increased by 40%, from 11 to 51%. Households with a child <six years old at the time of the survey, a proxy for those targeted, were significantly more likely to have received a campaign bed net (80.7 *vs* 35.2%, p < 0.001). LLIN ownership and use was equitable after the targeted campaign, with no significant differences by household wealth status.

However, the proportion of households with at least one LLIN per two people was still low after the first campaign phase, increasing from 8.5 to 25.9%.

**Conclusions:**

The first phase of the campaign led to substantial increases in both LLIN ownership and equitable use among children <five years in the Central region. However, access to an LLIN within the household was still low after the first phase of the campaign, indicating the need for the universal fill-in campaign.

## Background

Long-lasting, insecticide-treated bed nets (LLINs) are an important public health strategy for malaria prevention adopted by most countries with endemic malaria. In addition to serving as physical barriers between mosquito vectors and individual users, toxicity and repellency induced by the pyrethroid insecticide-impregnated in LLINs can have important community-wide effects on vector density [1-3], and LLINs have been shown to reduce the burden of malaria, especially among children <five years and pregnant women [4,5] who are most vulnerable to malaria. LLINs are also one of the most cost-effective interventions, particularly in areas of high-malaria transmission [6].

To increase coverage of LLINs, especially among the vulnerable groups, previous WHO guidelines focused on targeted provision to pregnant women and children <five years especially in areas of high malaria transmission. The Uganda National Malaria Control Programme (NMCP) adopted this policy in 2002 and pursued strategies that included free distribution to pregnant women through antenatal care visits, provision of subsidized nets through the private sector and sale of full-cost nets in the commercial sector. However, despite such endeavours, LLIN coverage in Uganda, as in other countries, fell short of the World Health Assembly resolution targets of 80% bed net coverage by 2010 [7]. Based on a Malaria Indicator Survey (MIS) conducted in 2009, LLIN ownership in Uganda was 46% nationwide, with the lowest ownership rate of 22% found in the Central region; LLIN usage among children <five years was even lower, at 32% nationwide and 11% in the Central region [8]. At this time, Uganda had not sponsored any national distribution campaigns, although donors and non-governmental organizations had supported local bed net distributions in areas in which they worked.

With funding from The Global Fund to Fight AIDS, Tuberculosis and Malaria in 2007, the NMCP decided to carry out its first targeted community mass distribution campaign, beginning in the Central region of Uganda, with the objective of increasing LLIN coverage among children <five years and pregnant women to at least 90% by the end of 2010. Before the campaign began, malaria researchers began to advocate for funding for universal distribution campaigns given benefits of universal bed net coverage [9], and WHO issued a position statement in 2007 supporting full coverage of bed nets for all people at risk of malaria [10]. The NMCP decided to implement the campaign in two phases, with the first phase targeting households with vulnerable populations (pregnant women or children <five years) and the second phase filling in the gaps to achieve universal coverage. The first phase began in spring 2010 in the Central region, which had the lowest bed net coverage at the time, as it had not benefitted from any local bed net campaigns previously.

The NMCP, working together with district health management teams and civil society organizations, distributed 1,481,050 LLINs in April and May 2010 in 13 districts of the Central region. Households were initially enumerated and registered by village health teams, and each household was eligible to receive one LLIN for each child under five years and for each pregnant woman. Pregnant women were identified by observation and/or the presence of an antenatal card. To estimate changes in LLIN ownership and usage after the first phase of the bed net distribution in the Central region of Uganda, a survey was conducted from January to February 2011 to compare with data from the 2009 MIS, which was conducted in November-December 2009.

## Methods

### Study design and sample size

**Figure 1:**
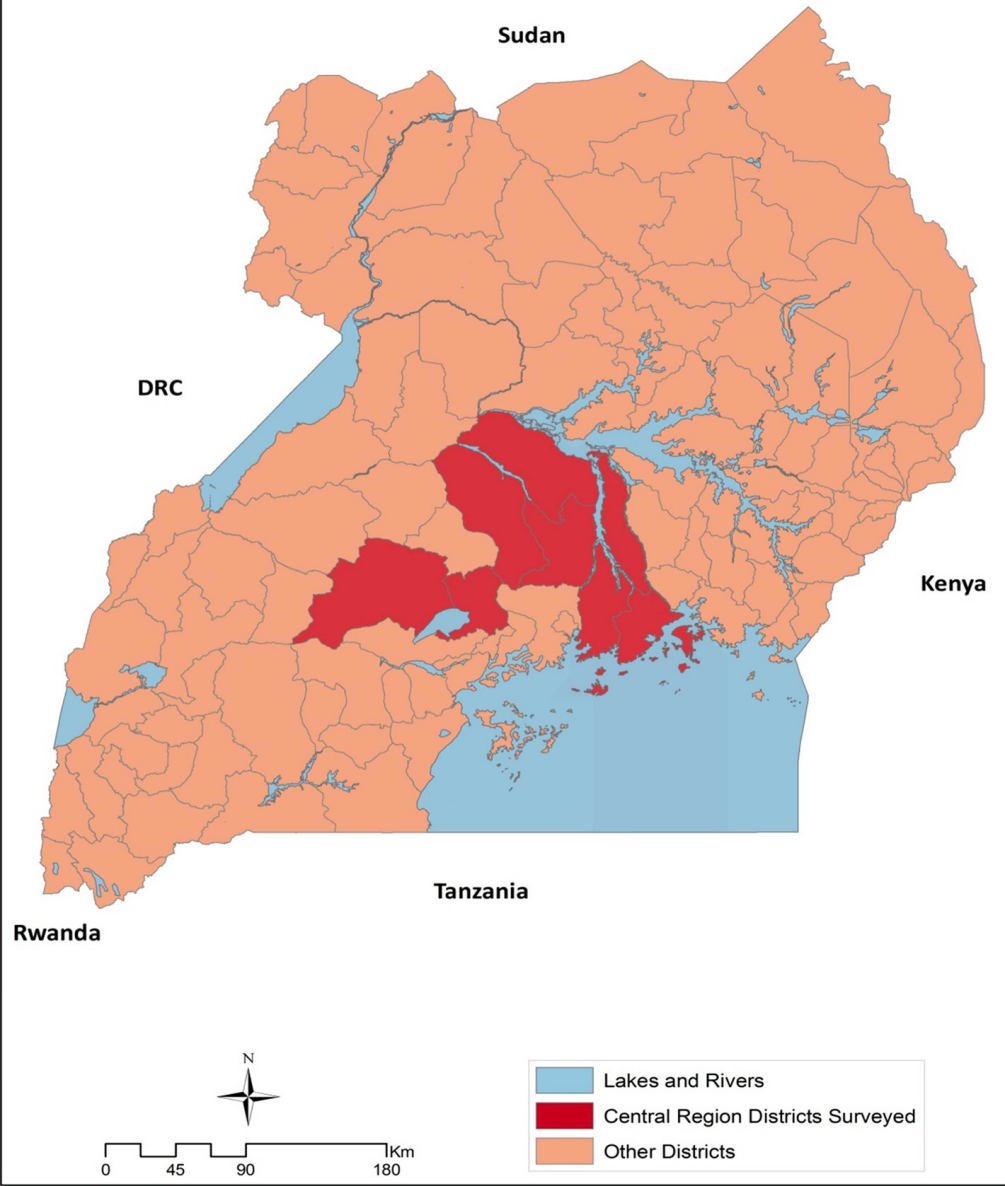
Map of Uganda showing the central region survey area.

A two-stage, cluster-sample, cross-sectional household survey was carried out in seven of the eight Central region districts chosen for the Central 2 region of the 2009 MIS (Figure 1); one district was excluded as it had recently received an NGO-supported universal coverage campaign. In the first sampling stage, 30 enumeration areas (EAs) located in the study districts were selected from a list of EAs used in the 2002 Uganda population census using probability proportionate to size sampling. Within each sampled EA, all households were listed and 20 were randomly sampled from the listing for inclusion in the survey. In each household, an adult household member aged ≥18 years, if possible the head of household, was asked to respond to the household questionnaire (see below) and all women aged 15-49 in selected households were asked to participate in the woman’s questionnaire.

Allowing for a 10% non-response rate and a design effect of 1.7, a sample size of 600 households was calculated to estimate net use within 5% and to assess changes in LLIN ownership and usage of 25% or greater relative to 2009 MIS Central region measures, translating to a prevalence ratio of 1.25 or greater, with 80% power.

### Study questionnaire**s** and variables

Two questionnaires were used in the survey: a household questionnaire and a women’s questionnaire for all women aged 15-49 years in selected households. Both instruments were based on the model MIS questionnaires developed by the Roll Back Malaria Partnership Monitoring and Evaluation Reference Group, as well as other questionnaires from previous surveys conducted in Uganda, including the 2006 Uganda Demographic and Health Survey (UDHS) and the 2009 Uganda MIS.

The questionnaires were translated into Luganda, the major local language commonly spoken in central Uganda. The questionnaires were pre-tested prior to the main data collection to assess the appropriateness of the wording of the questions and to verify the translations and skip patterns.

The household questionnaire was used to list all usual members and visitors in the selected households, inquire about bed net ownership and use, and gather information on household assets and characteristics. The women’s questionnaire was used to collect information from all women aged 15-49 years on background characteristics (age, education, literacy, employment), recent reproductive history, pre-natal care and preventive malaria treatment received during pregnancy for the most recent birth, treatment of fever among children under five and knowledge about malaria (causes, ways to avoid, types of medicines).

### Data collection

Training for survey teams lasted ten days and included didactic sessions on survey objectives, methodology and questionnaires, classroom role-play and mock interviews, field testing, and feedback to trainees. A total of three survey teams, each composed of three interviewers and one supervisor, carried out the survey over three weeks, between 17 January and 7 February, 2011. Survey teams spent up to two full days in each EA and made at least three attempts to interview each sampled household. The teams used hand-held computer tablets programmed using QDS software (Nova Research, Bethesda, Maryland, USA).

### Data analysis

Data were weighted using survey weights that accounted for the probability of selection at each stage as well as non-response at the household level. Analysis of key bed net ownership and use variables were adjusted for the complex survey design using the svy commands in Stata 11.0 (Statcorp, College Station, Texas, USA), which account for both the survey weights and clustering at EA level, using Taylor series linearization. Key variables from the 2011 data on bed net ownership and use were compared to the 2009 MIS data for the Central region to assess trends after the targeted distribution.

Principal components analysis (PCA) was used to create wealth scores for each household based on ownership of selected assets, dwelling characteristics, source of drinking water, sanitation facilities, and other characteristics related to a household’s socio-economic status [11]. Specific variables included in the PCA were: source of water, source of toilet, source of fuel, flooring material, wall material, roof material, electricity, and household ownership of a radio, cassette player, television, mobile telephone, refrigerator, table, chair, sofa, bed, cupboard, clock, watch, truck, bank account, and scooter. The PCA accounted for 18.6% of the variability of the first component, within the range found in other studies using a PCA approach to characterize wealth [12]. To assess the effectiveness of the campaign targeted to households with pregnant women and children under five years of age, households were defined as eligible if they had a child under six years of age at the time of the survey, approximately eight months after the campaign, which would also capture households with pregnant women at the time of the campaign. Since there was no data on eligibility of each household during the campaign, this variable is only a proxy for eligibility at the time of the bed net campaign.

For assessing predictors of LLIN use among children, bivariate and multivariate prevalence ratios from Poisson regression (with robust standard errors) were used, which have been shown to more accurately reflect risk than odds ratios when the outcome is common [13]. Predictors of bed net use that were significant at p < 0.10 in bivariate analyses were included in a multivariate model.

### Ethical approval

The protocol was reviewed and approved by the Centers for Disease Control and Prevention in Atlanta, GA and by the Committee for Human Research of the University of California San Francisco, USA. Ethical approval for the survey was also obtained from the Makerere University College of Health Sciences Research and Ethics Committee and the Uganda National Council of Science and Technology.

## Results

A total of 447 households were surveyed in the Central 2 region in the 2009 MIS, and 556 were surveyed in areas targeted for the bed net campaign during the 2011 survey. After the survey of 30 EAs was completed, it was discovered that five of the surveyed EAs were in areas of the Central region that were not targeted for the first phase of the bed net campaign, as they had recently received bed nets from NGOs. Therefore, responses from these five EAs were excluded from the analysis, yielding 458 households for analysis. Bed net ownership increased substantially between these two periods, from 40.7% of households owning any net in 2009 to 78.3% in 2011. LLIN ownership increased even more dramatically, from 22.3 to 69.2% (Table 1). Among eligible households, defined as households with a child <six years at the time of the survey, LLIN ownership was even higher, at 84.8%.

**Table 1:** Bed net ownership, bed net source and net use by children under five years, by date

**Variable**	**2009 MIS central region**	**2011 central region survey**	**95% confidence interval (CI)**
*Household characteristics*	*n = 447*	*n = 458*	
Owns any nets	40.7	78.3	[71.8, 83.7]
Mean number nets per household	0.7	1.9	[1.7, 2.1]
Owns 1+ LLIN	22.1	69.2	[62.0, 75.6]
Mean number LLINs per household	0.4	1.5	[1.3, 1.7]
At least 1 ITN per 2 people	8.5	25.9	[20.8, 31.6]
*All household members*	*n = 1,998*	*n = 2,300*	
Proportion of population with access to ITN in household*	3.6	22.1	[16.8, 28.5]
*Bed net source*	*n = 304*	*n = 870*	
Government health facility	14.1	9.9	[6.0, 15.9]
Shop/open market/hawker	48.7	25.4	[19.5, 32.3]
Project/NGO/Church	24.0	3.4	[1.8, 6.1]
Campaign	2.4	58.6	[50.0, 66.7]
Other	6.1	2.5	[1.6, 3.8]
Don't know	4.8	0.3	[0.1, 1.1]
*Children < five years old*	*n = 352*	*n = 426*	
Slept under any net	20.7	57.2	[48.9, 65.1]
Slept under LLIN	10.6	50.6	[43.9, 57.2]

Note: CIs account for cluster survey design using Taylor series linearization.

*Defined as the proportion of individuals who could have slept under an LLIN, assuming each LLIN is used by two people.

In 2009, the most common source of bed nets was the open market or shops, where nearly half of nets were purchased, but this decreased in 2011 to 25.4% of nets. In 2011, the majority of bed nets (58.6%) were reported to be from the 2010 campaign. Bed net use also increased substantially from only 10.6% of children <five years sleeping under an LLIN to more than half (50.6%) of children in 2011 (Table 1).

Access to LLINs within households, defined as one LLIN per two people [14], increased from 8.5% in 2009 to 25.9% in 2011, but remained low, with only about one-quarter of households having adequate access to LLIN (Table 1). This was even lower (3.6% in 2009 and 22.1% in 2011) when adequate access was defined as the proportion of the population within households having adequate access to LLINs.

Significantly more households that were eligible, according to the definition of having a child <six years at the time of the survey, received at least one bed net during the campaign, compared to households not eligible (80.3 *vs* 35.2%, p < 0.001) (Table 2). Eligible households received on average 1.6 bed nets from the 2010 campaign and non-eligible households 0.6, (p < 0.001). However, one-fifth of eligible households reported they did not receive a bed net, and more than one-third of non-eligible households receiving one or more campaign bed nets. Of the eligible households who did not receive a campaign bed net, 13.1% said they were not on the eligible list of households, and 18.0% reported being unaware of the campaign (Table 2).

**Table 2:** Campaign visits and nets received, by eligible household status

**Variable**	**All households**	**95% CI**	**Eligible households**	**95% CI**	**Non-eligible households**	**95% CI**	**p-value**
	*n = 458*		*n = 271*		*n = 187*		
Received pre-campaign visit	72.7	[63.1, 80.6]	80.2	[70.4, 91.6]	61.1	[50.0, 72.2]	0.001
Received post-campaign visit	10.0	[6.0, 16.1]	11.7	[6.3, 20.6]	7.5	[4.3, 12.2]	0.153
Number bed nets received							
None	37.4	[29.8, 45.9]	19.3	[10.5, 32.7]	64.8	[55.9, 72.8]	<0.001
One	25.0	[19.9, 30.8]	27.6	[20.7, 35.6]	21.2	[14.9, 29.1]	
Two	19.1	[15.2, 23.7]	26.3	[20.4, 33.3]	8.2	[5.1, 13.0]	
Three or more	18.4	[13.2, 25.2]	26.8	[19.2, 36.1]	5.8	[2.7, 11.7]	
Mean	1.2	[1.0, 1.4]	1.6	[1.3, 1.9]	0.6	[0.4, 0.7]	<0.001
Reasons for not receiving a bed net	*n = 166*		*n = 48*		*n = 113*		
Not on list/not eligible for bed net	45.1	[31.6, 59.4]	13.1	[4.2, 34.2]	59.6	[47.3, 70.7]	<0.001
None available/no bed nets left	4.1	[1.7, 9.6]	4.2	[0.8, 19.1]	4.0	[1.3, 11.9]	
Was not aware of campaign	10.9	[7.3, 16.0]	18.0	[8.9, 32.8]	7.7	[3.9, 14.9]	
Not home during distribution week	20.4	[11.8, 32.9]	32.6	[12.2, 62.9]	14.9	[8.7, 24.4]	
Did not know where to go/distribution location too far	6.2	[1.7, 20.5]	9.8	[1.9, 37.7]	4.6	[1.5, 13.0]	
Other/Don't know	13.1	[6.4, 25.6]	22.4	[7.9, 49.6]	9.2	[5.2, 15.9]	

Note: Eligible household defined as a household with either one or more children under six years old and/or a pregnant woman. CIs account for cluster survey design using Taylor series linearization.

Receipt of a campaign bed net was equitable. Wealth quintile was not related to receipt of a bed net among either eligible (p = 0.94) or non-eligible households (p = 0.74) (Table 3). Other socio-economic status variables, including presence of a woman in the household with either secondary school education or higher or who listened to the radio at least once per week, were also not related to receipt of a campaign bed net. The size of a household was significantly related to receipt of at least one campaign net, with each additional person in the household increasing the chances of receiving a bed net among eligible households by 2% (p = 0.01) and among non-eligible households by 17% (p < 0.001) (Table 3).

**Table 3:** Factors associated with receipt of at least one campaign net, by eligible household status

	**Eligible households (n = 271)**			**Non-eligible households (n = 187)**		
**Variable**	**Unadjusted prevalence ratio**	**95% CI**	**p-value**	**Unadjusted prevalence ratio**	**95% CI**	**p-value**
Wealth quintile	1.00*	[0.90, 1.12]	0.935	1.03*	[0.88, 1.20]	0.743
Number people in household	1.02**	[1.01, 1.04]	0.01	1.17**	[1.10, 1.24]	<0.001
Child 6-8 years				2.86	[1.90, 4.28]	<0.001
Woman in household with at least secondary school education	1.05	[0.82, 1.36]	0.673	0.92	[0.47, 1.81]	0.812
Woman in household who listens to radio at least once/week	1.43	[0.95, 2.17]	0.087	1.05	[0.38, 2.86]	0.928

*per unit increase.

**per one person increase.

Note: Eligible household defined as a household with either one or more children under six years old and/or a pregnant woman. CIs account for cluster survey design using Taylor series linearization.

Slightly more than half of children <five years slept under an LLIN the previous night (Table 1). The strongest predictors of LLIN use the previous night among children <five years were the number of LLINs in the household in relation to household size, as well as receipt of a 2010 campaign net (Table 4). With each additional LLIN per household member, the adjusted chance of a child <five years sleeping under an LLIN increased 2.45 fold (p = 0.053). Adjusting for the LLIN: household member ratio, children living in households that received a bed net from the 2010 campaign were 29% more likely to sleep under an LLIN the previous night than those in households that had not received a campaign bed net (p = 0.015). Models were also run using the household-level variable of possessing at least one LLIN per two people. This variable was also significant (adjusted prevalence ratio = 1.61, 95% CI: 1.16, 2.24) in a multivariate model with receipt of a campaign net (adjusted PR = 1.33, 95% CI: 1.07, 1.64).

**Table 4:** Individual- and household-level predictors of long-lasting, insecticidal-treated bed net use among children <five years

**Variable**	**Unadjusted prevalence ratio**	**95% CI**	**p-value**	**Adjusted prevalence ratio**	**95% CI**	**p-value**
Wealth quintile*	1.05	[0.94, 1.18]	0.331			
Knowledge that mosquitoes cause malaria	1.13	[0.79, 1.61]	0.488			
Mother never listens to radio	0.65	[0.34, 1.25]	0.183			
LLIN: household member ratio	2.61	[1.04, 6.54]	0.042	2.45	[0.99, 6.09]	0.053
Received a bed net in 2010 campaign	1.34	[1.07, 1.68]	0.013	1.29	[1.06, 1.59]	0.015

*1=poorest; 5=least poor.

## Discussion

This study showed that a targeted community distribution of LLINs substantially increased LLIN ownership and usage in the Central region of Uganda. Households owning at least one LLIN increased by 47%, from 22 to 69%, and LLIN ownership among eligible households was 84%, close to the campaign target of 90%.

Despite remaining gaps, the greatest gains in net usage have been seen after mass community distributions of bed nets [15-19]. Furthermore, such distribution tends to help close equity gaps in bed net ownership and use that existed prior to campaigns [20,21]. A previous study in Uganda showed that strategies such as socially marketed, subsidized nets alone led to overall low levels of LLIN use among children and inequity in bed net ownership in Uganda [22]. Another study indicated that prior to the campaign, bed net use in Uganda was inequitable, with children in wealthy households significantly more likely to sleep under a bed net the previous night [23]. These results showed that wealth was not associated with either household receipt of a campaign net or with children <five years sleeping under an LLIN the previous night. The only factors this survey found to be significantly associated with LLIN use were the availability of LLINs in the household, measured by the LLIN: household member ratio or access to one LLIN per two people, and, more importantly, receipt of a campaign bed net.

Targeting of bed nets to eligible households was reasonably effective, with 81% of eligible households receiving a campaign bed net compared to 35% of non-eligible households receiving a campaign net. One notable limitation of this study is that the measure of ‘eligibility’ is a proxy, since there was no actual measure of eligibility at the time of the campaign. However, despite potential measurement error introduced by this proxy indicator, the under-coverage of ‘eligible’ households and leakage to non-eligible households is substantial enough to indicate imperfect targeting of households during the 2010 campaign.

Despite large increases in household LLIN ownership LLIN and usage among children <five years old, which was the target of the first phase of the campaign, adequate LLIN access within the household was still very low after the first campaign phase. Nearly three-quarters of household did not have at least one LLIN per two people, which is the current standard for universal LLIN access.

## Conclusions

These survey findings show that the first phase of Uganda’s LLIN campaign, which targeted bed nets to households with children <five years and pregnant women, led to substantial increases in bed net ownership and equitable bed net use among children <five years in the Central region. Utilization of LLINs by children <five years increased nearly five-fold, but more work remains to be done to meet the NMCP’s target of 80% for both household ownership of at least two LLINs and 80% of children <five years sleeping under one by 2015. The second phase of the national bed net campaign in Uganda, designed to achieve universal bed net coverage, should help move closer towards that target, and progress should be closely monitored. In addition, continuous distribution strategies, for example through antenatal care, child vaccination clinics, and subsidies for socially marketed nets, among other approaches, should help maintain high bed net coverage in between mass campaigns [10,17].

## Abbreviations

EA: Enumeration area; LLIN: Long-lasting, insecticide-treated bed nets; NGO: Non-governmental organization; NMCP: National Malaria Control Programme; UDHS: Uganda demographic and health survey; WHO: World Health Organization; PCA: Principal component analysis.

## Competing interests

The authors declare that they have no competing interests.

## Authors’ contributions

AY, SN, MK, SF, and GD conceived and designed the study. HW led the data collection, with assistance from LS, RK, DR, and AS. LS led the data analysis with input from all authors. All authors participated in the writing of the manuscript and have read and approved the final manuscript.
